# Trends and spatial distribution of pediatric second-dose COVID-19 vaccination coverage: a temporal analysis, Brazil, 2022-2023

**DOI:** 10.1590/S2237-96222026v35e20250054.en

**Published:** 2026-02-27

**Authors:** Kassya Fernanda Freire Lima, Marta Silva de Santana, Girlane Caroline Pereira Santos, Pablo Nascimento Cruz, Thaysa Goes Trinta Abreu, Lívia Maia Pascoal, Rosângela Fernandes Lucena Batista, Rosilda Silva Dias, Bruno Luciano Carneiro Alves de Oliveira

**Affiliations:** 1 Universidade Federal do Maranhão, Departamento de Enfermagem, São Luís, MA, Brazil

**Keywords:** Vaccination, Pediatrics, COVID-19 Vaccines, Vaccination Coverage, Time Series Studies, Vacunación, Pediatría, Vacunas contra la COVID-19, Cobertura de Vacunación, Estudios de Series Temporales

## Abstract

**Objective:**

To investigate the trend and spatial distribution of pediatric second-dose COVID-19 vaccination coverage among children aged 5 to 11 years in Brazil, in 2022 and 2023.

**Methods:**

This was a time series analysis study using vaccination and target population data extracted from the Ministry of Health Vacinômetro platform. The second-dose coverage trend was estimated by joinpoint regression (segmented regression), while spatial distribution was analyzed using choropleth maps to identify regional variations between the Brazilian states.

**Results:**

Average monthly percentage change (AMPC) in vaccination coverage in Brazil as a whole remained stable during the period (AMPC -8.30%), with significant geographical heterogeneity. The Northern region (AMPC 5.76%) had a rising trend, in particular in the states of Acre (AMPC 23.42%) and Amazonas (AMPC 18.26%). Falling trends were observed in the Southeast region (AMPC -8.30%) as well as in specific states, such as Mato Grosso do Sul (AMPC -4.74%), Pará (AMPC -4.71%) and Rio de Janeiro (AMPC -4.27%). Fourteen states (51.9%) had a stable trend. The time series revealed initial increase in 2022, followed by deceleration and stabilization in 2023.

**Conclusion:**

A significant and uneven variation in pediatric second-dose COVID-19 vaccination coverage was observed between 2022 and 2023. The results highlight the urgent need for targeted strategies to increase vaccination adherence, especially in areas with lower coverage.

## Resumen

**Objetivo:**

Investigar la tendencia y la distribución espacial de la cobertura de vacunación pediátrica contra la COVID-19, con énfasis en la segunda dosis, en niños de 5 a 11 años en Brasil en 2022 y 2023.

**Métodos:**

Análisis de series temporales con datos de vacunación y población objetivo extraídos de la plataforma Vacinômetro del Ministerio de Salud. La tendencia en la cobertura de la segunda dosis se estimó mediante regresión de puntos de inflexión (regresión segmentada), mientras que la distribución espacial se analizó mediante mapas de coropletas para identificar variaciones regionales y entre unidades federativas (UF).

**Resultados:**

La variación porcentual mensual promedio (VPMP) en la cobertura de vacunación en el país se mantuvo estable durante el período (VPMP -8,30%), con una heterogeneidad geográfica significativa. La región Norte (VPMP 5,76%) mostró una tendencia ascendente, con énfasis en las UF de Acre (VPMP 23,42%) y Amazonas (VPMP 18,26%). Se observaron tendencias decrecientes en la región Sudeste (VPMP -8,30%) y en UF específicas, como Mato Grosso do Sul (VPMP -4,74%), Pará (VPMP -4,71%) y Río de Janeiro (VPMP -4,27%). Catorce estados (51,9%) mostraron una tendencia estable. La serie temporal reveló un crecimiento inicial en 2022, seguido de una desaceleración y estabilización en 2023.

**Conclusión:**

Se observó una variación significativa y desigual en la cobertura de vacunación pediátrica contra la COVID-19, con énfasis en la segunda dosis, entre 2022 y 2023. Los resultados resaltan la urgencia de implementar estrategias específicas para aumentar la adherencia a la vacunación, especialmente en áreas con menor cobertura.

**Ethical aspects**

This research used public domain anonymized databases.:

## Introduction

The COVID-19 pandemic was one of the biggest public health crises in recent history (1,2). In Brazil, the disease spread rapidly throughout the country, resulting in one of the highest morbidity and mortality rates in the world. From January to October 2022, the country recorded one death per day among children aged 6 months to 5 years diagnosed with COVID-19, totaling 314 deaths in this age group during that period (3,4).

COVID-19 vaccination played a fundamental role in reducing severe cases and deaths, proving to be an effective strategy for protecting the population and addressing the health emergency (5,6). Evidence indicated its benefits in all age groups, such as decreased transmission, case severity and risk of new variants (4,7). However, vaccine hesitancy represented a considerable obstacle in controlling the pandemic (8).

Brazil has one of the largest and most comprehensive immunization programs in the world, although some challenges regarding vaccination, especially against COVID-19, are still faced. Following approval and implementation of some COVID-19 vaccines, a lot of misinformation emerged regarding their efficacy and safety (9). Socioeconomic factors and those related to health service infrastructure may also have influenced vaccination coverage (4).

Between January 2021 and December 2022, Brazil administered 448.2 million doses of vaccines, distributed between first, second and third doses. Specifically among children aged 5-11 years, by December 2022, less than half of this population (46%) had received the first two doses of the vaccine (10). Considering the significant disparities in vaccination coverage in Brazil, this study aimed to investigate the trend and spatial distribution of pediatric second-dose COVID-19 vaccination coverage among children aged 5-11 years in Brazil in 2022 and 2023.

## Methods

### Design and setting

This is a time series analysis study that assessed pediatric second-dose COVID-19 vaccination coverage, in order to identify temporal trends and spatial patterns in Brazil by state and macro-region in the period 2022-2023.

The units of analysis were the 26 Brazilian states (Acre, Alagoas, Amapá, Amazonas, Bahia, Ceará, Espírito Santo, Goiás, Maranhão, Mato Grosso, Mato Grosso do Sul, Minas Gerais, Pará, Paraíba, Paraná, Pernambuco, Piauí, Rio de Janeiro, Rio Grande do Norte, Rio Grande do Sul, Rondônia, Roraima, Santa Catarina, São Paulo, Sergipe and Tocantins) and the Federal District, as well as Brazil’s five macro-regions (North, South, Southeast, Midwest and Northeast). 

### Participants

The study analyzed aggregated secondary data on second doses of the Pfizer-BioNTech monovalent pediatric vaccine (Comirnaty pediatric vaccine) against COVID-19 administered to children aged 5-11 years in Brazil.

### Variables

The main variable of the study was the vaccination coverage rate, obtained by the ratio between the number of individuals who received the second dose and the target population, multiplied by 100 to express the result as a percentage (13).

The rates were calculated by month, totaling 24 analysis points, between January 2022 and December 2023. The target population used as the denominator was based on eligible children aged 5-11 years, according to the criteria established by the National Immunization Program.

We took into consideration the guidelines of the National Operational Plan for COVID-19 Vaccination (15), whereby vaccination was staggered, with initial prioritization of children with comorbidities, Indigenous and quilombola children and, subsequently, children without comorbidities, in the following age groups in descending order: 10-11 years, 8-9 years, 6-7 years and 5 years (15).

The database used in this study provided the total population according to this staggered vaccination procedure, in order to ensure the adequacy of the denominator according to the eligibility phases (14,15).

### Data source and measurement 

Vaccination and target population data were extracted from the Ministry of Health Vacinômetro platform (14).

The platform was developed by the Department of Monitoring, Evaluation and Dissemination of Strategic Health Information, linked to the Information and Digital Health Secretariat, in collaboration with the Department of Immunization and Vaccine-Preventable Diseases, part of the Health and Environmental Surveillance Secretariat. 

The data source for the cartographic base (territorial grids/spatial files) used to create the choropleth maps was obtained using its 2021 version (16). All data used in this research, referring to the period from January 2022 to December 2023, were collected in September 2024.

### Statistical methods

The extracted data were consolidated using Microsoft Excel version 365. Vaccination coverage rates were calculated monthly for each Brazilian federative unit and macro-region between January 2022 and December 2023.

The rates were exported for segmented regression (Joinpoint Regression Program version 5.2.0). This software has been widely used for trend analysis and identification of inflection points in time series, especially in epidemiological studies (17). It has made it possible to identify periods of significant changes in rates and to quantify the magnitude of these changes over time (18).

The dependent variable (Y) was the monthly vaccination coverage rate of the vaccine administered to the 5-11 year old population, while the month was taken to be the independent variable (X).

The analysis was performed using segmented regression with log transformation, employing the Monte Carlo permutation method to verify whether multiple segments explained the model better than a single straight line.

Monthly percentage change (MPC) was calculated with a 95% confidence interval (95%CI). At the end of the analysis, the overall trend of each series was summarized by the average monthly percentage change (AMPC) between the segments, with their respective 95%CI (20).

The model was fitted from zero (one segment) to four inflection points (three segments), considering a significance level of 5% to test the hypothesis that the MPCs were statistically different from zero. Results with p-value<0.005 were considered significant, while negative coefficients (AMPC/MPC) indicated a falling trend, and positive coefficients indicated a rising trend (21).

We used RStudio software, version 2024.09 (22), for graphical and comparative demonstration. Choropleth maps were built using QGIS software, version 3.34.8 (23), to visualize the distribution of vaccination coverage rates and identify geographical variations, according to state and macro-region. The simple arithmetic average of the monthly vaccination coverage rates was calculated, taking the sum of the monthly rates and then dividing them by the complete period (24 months).

## Results

**Figure 1:**
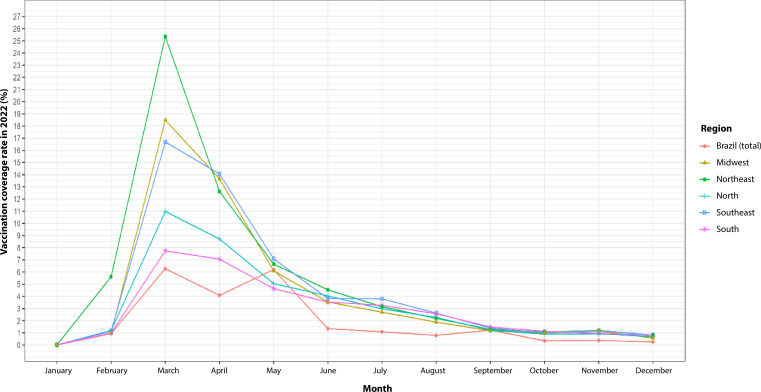
Second-dose COVID-19 vaccination coverage (%) among children aged 5-11 years. Brazil and its macro-regions, 2022 (n=57,506,832)

The results of this study indicated that, throughout 2022, the rate of childhood second-dose COVID-19 vaccination coverage varied between the Brazilian regions—Midwest, Northeast, North, Southeast and South—apart from the national total. This rate peaked in March in all regions of Brazil. The Northeast presented the highest rate, at 26.0%, while the Midwest and North reached peaks of 18.0% and 16.0%, respectively. The Southeast and South registered peaks of 10.0% and 8.0%. After March, there was a gradual decline in rates in all regions. Compared to the other regions, the Southeast and Southern regions maintained higher vaccination coverage after the initial peak (Figure 1).

In 2023, childhood second-dose COVID-19 vaccination coverage in Brazil remained below 1.0% in all months and regions, with the exception of the Southern region, which surpassed this mark in March, reaching 10.0%. The North and Northeast regions showed similar behavior, but with lower rates, ranging between 0.5% and 0.8%, compared to the South and Southeast, which presented higher rates, with the Southeast reaching 7.5% in March. The Midwest approached the national average in several aspects, with rates ranging from 0.9% to 1.0%, reinforcing an intermediate pattern. Overall, all regions maintained low vaccination coverage levels, especially when compared to the previous year.

Segmented regression trend analysis indicated stability in average monthly percentage change (AMPC -8.3%; 95%CI -21.6; 0.3) in second-dose COVID-19 vaccination coverage among the 5-11 year old population between 2022 and 2023.

**Figure 2:**
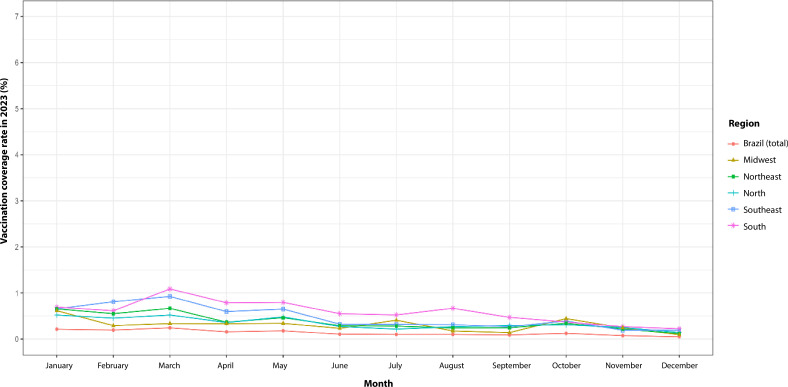
Second-dose COVID-19 vaccination coverage (%) among children aged 5-11 years. Brazil and its macro-regions, 2023 (n=57,506,832)

Regional data analysis revealed distinct patterns between the Brazilian macro-regions. A statistically stable trend in vaccination coverage was observed in the Northeast region (AMPC 5.2%; 95%CI -0.4; 11.4), indicating absence of significant increase. In the North, a rising trend was observed (AMPC 5.7%; 95%CI 1.8; 13.0). In the Midwest (AMPC 2.0%; 95%CI -0.8; 6.7) and South (AMPC 5.8%; 95%CI -0.1; 13.3), the trend was stable. In the Southeast, there was a falling trend (AMPC -8.30%; 95%CI -11.2; -5.9), contrary to the pattern observed in the other regions (Table 1).

**Table 1:** Average monthly percentage change (AMPC) and 95% confidence interval (95%CI) of second-dose COVID-19 vaccination coverage among children aged 5-11 years. Brazil, macro-regions and federative units, 2022-2023 (n=24)

Variable	Average vaccination coverage 2022 (%)	Average vaccination coverage 2023 (%)	AMPC	Lower (95%CI)	Upper (95%CI)	p-value	Trend
**Northeast**	4.4	0.4	5.2	-0.4	11.4	0.066	Stable
Alagoas	3.5	0.2	28.1	2.2	58.9	0.025	Rising
Bahia	4.2	0.4	4.0	-1.6	11.5	0.143	Stable
Ceará	5.0	0.5	5.2	0.5	14.6	0.025	Rising
Maranhão	3.0	0.4	7.1	-6.3	18.9	0.094	Stable
Paraíba	4.9	0.6	11.7	-0.7	16.4	0.068	Stable
Pernambuco	2.6	0.2	11.8	-8.4	34.2	0.107	Stable
Piauí	6.0	0.5	3.2	-1.8	10.2	0.237	Stable
Rio Grande do Norte	4.5	0.3	0.1	-3.3	3.9	0.944	Stable
Sergipe	5.0	0.6	10.2	4.7	17.6	0.032	Rising
**North**	2.8	0.5	5.7	1.8	13.0	0.002	Rising
Acre	2.7	0.5	23.4	8.4	47.4	0.010	Rising
Amapá	3.5	0.9	12.1	5.5	24.5	<0.001	Rising
Amazonas	3.5	1.0	18.2	10.0	43.3	0.010	Rising
Pará	2.7	0.4	-4.7	-10.2	-0.6	<0.024	Falling
Rondônia	1.9	0.3	0.6	-3.8	6.8	0.767	Stable
Roraima	1.4	0.4	16.6	10.0	31.5	0.001	Rising
Tocantins	2.1	0.2	8.1	-6.3	24.6	0.106	Stable
**Midwest**	3.2	0.3	2.0	-0.8	6.7	0.194	Stable
Distrito Federal	4.5	0.5	-0.0	-4.1	5.4	0.835	Stable
Goiás	3.3	0.3	8.8	3.0	18.5	0.001	Rising
Mato Grosso	2.3	0.2	3.5	-2.3	12.1	0.208	Stable
Mato Grosso do Sul	3.1	0.3	-4.7	-8.3	-0.2	0.040	Falling
**Southeast**	5.3	0.3	-8.3	-11.2	-5.9	<0.001	Falling
Rio de Janeiro	4.2	0.2	-4.2	-7.9	-0.7	0.027	Falling
Espírito Santo	4.2	0.6	8.0	-0.6	22.3	0.064	Stable
Minas Gerais	5.1	0.3	5.5	-1.3	15.2	0.081	Stable
São Paulo	5.9	0.3	12.7	-15.8	-10.4	<0.001	Rising
**South**	4.2	0.3	5.8	-0.1	13.3	0.058	Stable
Paraná	3.4	0.2	3.9	-2.4	11.7	0.230	Stable
Santa Catarina	2.8	0.1	3.6	-4.1	10.7	0.228	Stable
Rio Grande do Sul	4.4	0.3	9.3	3.1	16.2	0.001	Rising
**Brazil**	1.9	0.1	-8.3	-21.6	0.3	0.057	Stable

Ten states (37.0%) stood out as having rising trends: Alagoas (AMPC 28.1%; 95%CI 2.2; 58.9), Acre (AMPC 23.4%; 95%CI 8.4; 47.4), Amazonas (AMPC 18.2%; 95%CI 10.0; 43.3), Roraima (AMPC 16.6%; 95%CI 10.0; 31.5), Amapá (AMPC 12.1%; 95%CI 5.5; 24.5), Sergipe (AMPC 10.2%; 95%CI 4.7; 17.6), Rio Grande do Sul (AMPC 9.3%; 95%CI 3.1; 16.2), Goiás (AMPC 8.8%; 95%CI 3.0; 18.5), Tocantins (AMPC 8.1%; 95%CI -6.3; 24.6) and Ceará (AMPC 5.2%; 95%CI 0.5; 14.6). Three states showed a falling trend (11.1%): Mato Grosso do Sul (AMPC -4.7%; 95%CI -8.3; -0.2), Pará (AMPC -4.7%; 95%CI -10.2; -0.6) and Rio de Janeiro (AMPC -4.2%; 95%CI -7.9; -0.7). The other 14 states showed a stable trend throughout the period (51.9%).

**Figure 3:**
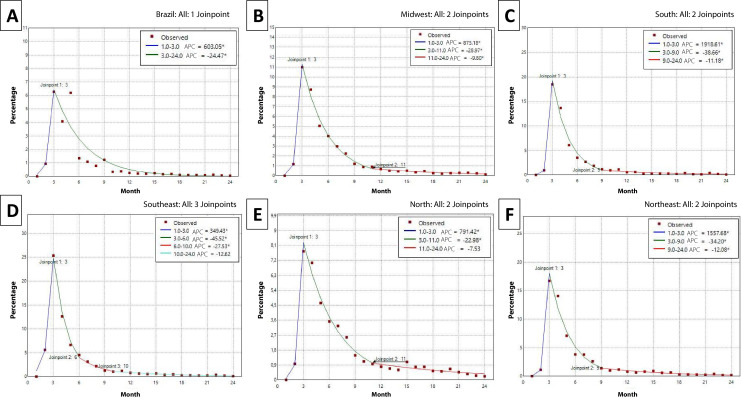
Annualized Percentage Change (APC) and 95% confidence interval of second-dose COVID-19 vaccination coverage among children aged 5-11 years: (A) Brazil; (B) Midwest; (C) South; (D) Southeast; (E) North; (F) Northeast. Brazil and its macro-regions, 2022-2023 (n=24)

The MPC analysis revealed marked variations in vaccination coverage over time in the different regions of Brazil, with a common pattern of initial increase followed by sharp falls and subsequent stabilization (Figure 3). Over the 24-month period, the greatest variations occurred in the first months of 2022, with a significant increase followed by a rapid reduction in rates, which stabilized at low levels by 2023.

In Brazil, the first segment showed a high MPC of 603.0% (95%CI 48.6; 2,439.5) from January to March 2022, followed by a sharp deceleration of 24.4% (95%CI -30.7; -22.1) throughout the remainder of the time series. In the North and Northeast, the initial MPC values ​​were high, with 791.4% (95%CI 540.2; 1,929.1) in the North and 1,557.6% (95%CI 825.3; 3,275.1) in the Northeast, followed by a drop in the North (-22.9%; 95%CI -29.1; -20.1) and in the Northeast (-34.2%; 95%CI -38.8; -31.1). Following this, in the North, there was stability in the last months (annualized percentage change [APC] -7.5; 95%CI -12.4; 1.3) and, in the Northeast, the decline continued (MPC -12.0%; 95%CI -16.6; -7.3), although with less intensity.

In the Midwest, initial MPC was also high (875.1%; 95%CI 690.8; 1,615.8), followed by a gradual deceleration (MPC -28.9; 95%CI -31.9; -27.2) and a slower pace at the end of the segment (MPC -9.8%; 95%CI -13.9; -4.1). In the Southeast, there were 4 segmented regressions, with an initial increase of 349.4% (95%CI 293.3; 477.9), followed by a sharp drop in the third and sixth months (MPC -45.5%; 95%CI -48.6; -42.3) and from the sixth to the tenth month (MPC -27.5%; 95%CI -33.9; -13.5), with stabilization at the end of the series (APC -12.6%; 95%CI -22.3; 5.8).

In the South, high growth was observed (MPC 1,918.6%; 95%CI 1,079.5; 4,821.8) and then rapid deceleration (MPC -38.6; 95%CI -43.5; -35.4), being weaker at the end (MPC -11.1%; 95%CI -16.5; -5.3), similar to other regions.

**Figure 4:**
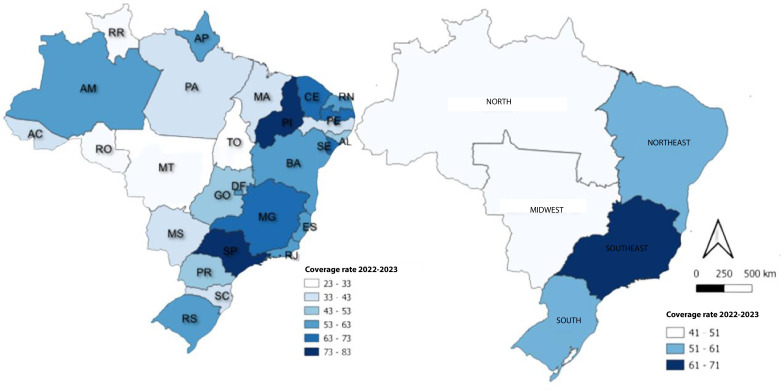
Spatial distribution of second-dose COVID-19 vaccination coverage (%) among children aged 5-11 years: (A) Federative Units; (B) Brazilian macro-regions. Brazil, 2022-2023 (n=57,506,832)

The Southeast region presented the highest second-dose COVID-19 vaccination coverage rates for children aged 5-11 years (61.0%-71.0%), followed by the Northeast and South regions, with intermediate rates (51.0%-61.0%). The lowest vaccination coverage rates were recorded in the Northern and Midwest regions. In the Northern region, rates ranged from 1.5% to 4.0%, while in the Midwest region, rates fluctuated between 2.0% and 4.5%. Piauí and São Paulo registered the highest rates (73.0%-83.0%) among the states, followed by Minas Gerais, Ceará, Pernambuco and Alagoas (63.0%-73.0%). Mato Grosso, Tocantins, Roraima and Rondônia presented the lowest rates (23.0%-33.0%) (Figure 4).

## Discussion

The analysis of pediatric second-dose COVID-19 vaccination coverage in Brazil in 2022 and 2023 revealed a scenario of significant geographical and temporal heterogeneity, despite national stability in the AMPC. A marked increase in coverage was observed in the Northern region, particularly in the states of Acre and Amazonas. In contrast, the Southeast region and some specific states (Rio de Janeiro, Mato Grosso do Sul and Pará) showed a falling trend.

This study had limitations inherent to the use of secondary data, which may be subject to inconsistencies, incompleteness and possible recording biases, which can lead to discrepancies in relation to actual vaccination coverage rates. Phased implementation of pediatric vaccination in Brazil, although taken into consideration when adjusting the population denominator, may have influenced the initial rates and affected temporal comparability between the states. Because this was a descriptive observational study with aggregated data, it was not possible to perform causal inferences or analyses at the individual level.

The geographical heterogeneity found in pediatric vaccination coverage corroborates other identified distinct patterns over time and between territories (15). Historically, the Northern region has the lowest coverage, followed by areas in the Northeast and Midwest (10,24). Up to December 2022, nine states were identified with vaccination coverage below 70% (located in the North and Northeast regions), recognized as having greater socioeconomic vulnerabilities (4).

Low coverage in these regions has been linked to factors such as lower human development indices, difficulties in accessing health services, large health service catchment areas and low population density, especially in municipalities in the Northern region (24,4,30). These structural inequalities contribute to fluctuations in indicators over time and reflect historical disparities in access to and administration of vaccines, in contrast to the South and Southeast regions, which traditionally have better vaccination coverage rates (30).

Evidence of stable or falling rates in certain regions highlights the urgency of understanding factors that limit vaccine adherence. Vaccine hesitancy, often exacerbated by the spread of misinformation, constitutes a significant obstacle (10). Factors such as political preferences, following certain social media and parental sociodemographic characteristics (age, income, education, religious affiliation) directly influence vaccine acceptance, with lower adherence observed among groups aligned with anti-vaccine stances (29).

Despite these challenges, it is crucial to stress the importance of pediatric vaccination. Immunization reduces the risks of severe COVID-19 cases, hospitalizations and deaths, including complications such as pediatric multisystem inflammatory syndrome and long COVID (25-28). Vaccination contributes to herd immunity, protection of vulnerable groups, as well as a safe return to daily activities, which alleviates the burden on health systems. Integrating COVID-19 vaccination with routine immunization programs and using incentives, such as counseling by health professionals, helps boost adherence (24).

The temporal analysis demonstrated a pattern of rapid growth in childhood immunization at the beginning of the COVID-19 vaccination campaign in 2022, followed by a sharp drop and stabilization at low levels throughout 2023. This behavior may be related to the start of the campaign, which took place under the coordination of the Ministry of Health, according to the guidelines of the National Plan for Operationalizing COVID-19 Vaccination. The strategy was nationwide, with provision for the distribution of doses to the states and municipalities, but its implementation varied according to local logistical capacity, social adherence and the structure of primary health care, resulting in different coverage rates between the states (14,15).

It is important to ensure more concrete actions to reverse the low vaccination coverage found in 2022 and 2023, such as strengthening regionalized educational campaigns, extending the opening hours of vaccination centers, encouraging the involvement of schools and greater integration between different levels of government, in order to guarantee equitable supply and access to childhood vaccination (4,6,24,29,30).

This study revealed a complex and heterogeneous scenario of pediatric second-dose COVID-19 vaccination coverage in Brazil, with divergent regional trends and a temporal pattern of deceleration after the initial advance. These findings are of great relevance for the planning of public policies, which must consider variations in order to strengthen childhood immunization and ensure the protection of this vulnerable population.

## Data Availability

The databases used for analysis in this research are available at: https://doi.org/10.6084/m9.figshare.29251784. The vaccination and target population data were extracted from the Ministry of Health Vacinômetro platform: https://infoms.saude.gov.br/extensions/SEIDIGI_DEMAS_Vacina_C19/SEIDIGI_DEMAS_Vacina_C19.html.
