# Supplementary material for: Trends and spatial distribution of pediatric second-dose COVID-19 vaccination coverage: a temporal analysis, Brazil, 2022-2023
Source: Epidemiol Serv Saude. 2026 Feb 27;35:e20250054. doi: 10.1590/S2237-96222026v35e20250054.en (PMC12948298; doi:10.1590/S2237-96222026v35e20250054.en)
Supplement: Translation

# Translation: Tendência e distribuição espacial da cobertura vacinal pediátrica da segunda dose contra a covid-19: análise temporal, Brasil, 2022-2023

**DOI:** 10.1590/S2237-96222026v35e20250054.pt

**Authors:** Kassya Fernanda Freire Lima, Marta Silva de Santana, Girlane Caroline Pereira Santos, Pablo Nascimento Cruz, Thaysa Goes Trinta Abreu, Lívia Maia Pascoal, Rosângela Fernandes Lucena Batista, Rosilda Silva Dias, Bruno Luciano Carneiro Alves de Oliveira

**Affiliations:** 1 Universidade Federal do Maranhão, Departamento de Enfermagem, São Luís, MA, Brasil

**Keywords:** Vacinação, Pediatria, Vacinas contra Covid-19, Cobertura Vacinal, Estudos de Séries Temporais

## Abstract

**Objetivo:**

Investigar a tendência e a distribuição espacial cobertura vacinal pediátrica contra a covid-19, com foco na segunda dose, em crianças de 5-11 anos no Brasil em 2022 e 2023.

**Métodos:**

Análise de série temporal com dados de vacinação e da população-alvo extraídos da plataforma Vacinômetro, do Ministério da Saúde. A tendência da cobertura da segunda dose foi estimada por regressão por pontos de inflexão (regressão segmenta), enquanto a distribuição espacial foi analisada por meio de mapas coropléticos, a fim de identificar variações regionais e entre unidades federativas (UF).

**Resultados:**

A variação percentual média mensal (VPMM) da cobertura vacinal no país manteve-se estável no período (VPMM -8,30%), com expressiva heterogeneidade geográfica. A região Norte (VPMM 5,76%) apresentou tendência crescente, com destaque para UF do Acre (VPMM 23,42%) e do Amazonas (VPMM 18,26%). Observaram-se tendências decrescentes na região Sudeste (VPMM -8,30%) e em UF específicas, como Mato Grosso do Sul (VPMM -4,74%), Pará (VPMM -4,71%) e Rio de Janeiro (VPMM -4,27%). Quatorze UF (51,9%) apresentaram tendência estável. A série temporal revelou crescimento inicial em 2022, seguida de desaceleração e de estabilização em 2023.

**Conclusão:**

Foi observada uma variação significativa e desigual na cobertura vacinal pediátrica contra a covid-19, com foco na segunda dose, entre 2022 e 2023. Os resultados destacam a urgência de estratégias direcionadas para aumentar a adesão à vacinação, especialmente em áreas com menor cobertura

**Aspectos éticos**

Esta pesquisa utilizou bancos de dados de domínio público e anonimizados.:

## Introdução

A pandemia de covid-19 foi uma das maiores crises de saúde pública da história recente (1,2) severe/critical n=66. No Brasil, houve rápida disseminação da doença por todo o país, o que resultou em uma das mais elevadas taxas de morbimortalidade no mundo. De janeiro a outubro de 2022, o país registrou um óbito por dia entre crianças de 6 meses a 5 anos de idade diagnosticadas com covid-19, o que totalizou 314 óbitos nessa faixa etária no período (3,4).

A vacinação contra a covid-19 desempenhou papel fundamental na redução de casos graves e de óbitos, mostrando-se estratégia eficaz para proteger a população e enfrentar a emergência sanitária (5,6)a global public dataset that tracks the scale and rate of the vaccine rollout across the world. This dataset is updated regularly and includes data on the total number of vaccinations administered, first and second doses administered, daily vaccination rates and population-adjusted coverage for all countries for which data are available (169 countries as of 7 April 2021. Evidências indicaram seus benefícios em todas as faixas etárias, como a diminuição da transmissão, da gravidade dos casos e do risco de novas variantes (4,7). Porém, a hesitação vacinal representou um obstáculo considerável no controle da pandemia (8).

O Brasil possui um dos maiores e mais completos programas de imunização do mundo, embora alguns desafios referentes à vacinação, especialmente contra a covid-19, ainda sejam enfrentados. Com a aprovação e a implementação de algumas vacinas contra a covid-19, surgiram muitas desinformações sobre a sua eficácia e a sua segurança (9). Fatores socioeconômicos e relacionados à estrutura de saúde também podem ter influenciado a cobertura vacinal (4).

Entre janeiro de 2021 e dezembro de 2022, o Brasil administrou 448,2 milhões de doses de vacinas, que foram distribuídas entre a primeira, a segunda e a terceira doses. Especificamente entre as crianças de 5-11 anos, até dezembro de 2022, menos da metade dessa população (46%) recebeu as duas primeiras doses da vacina (10). Ao considerar as disparidades significativas da cobertura vacinal no Brasil, este estudo teve por objetivo investigar a tendência e a distribuição espacial cobertura vacinal pediátrica contra a covid-19, com foco na segunda dose, em crianças de 5-11 anos no Brasil em 2022 e 2023.

## Métodos

### Delineamento e contexto

Trata-se de uma análise de série temporal que avaliou a taxa de cobertura da segunda dose da vacina pediátrica contra a covid-19, a fim de identificar tendências temporais e padrões espaciais no Brasil por unidade federativa (UF) e macrorregião no período 2022-2023.

As unidades de análise foram as 26 UF brasileiras (Acre, Alagoas, Amapá, Amazonas, Bahia, Ceará, Espírito Santo, Goiás, Maranhão, Mato Grosso, Mato Grosso do Sul, Minas Gerais, Pará, Paraíba, Paraná, Pernambuco, Piauí, Rio de Janeiro, Rio Grande do Norte, Rio Grande do Sul, Rondônia, Roraima, Santa Catarina, São Paulo, Sergipe e Tocantins) e o Distrito Federal, além das cinco macrorregiões do Brasil (Norte, Sul, Sudeste, Centro-Oeste e Nordeste). 

### Participantes

O estudo analisou dados secundários agregados de segundas doses da vacina pediátrica monovalente Pfizer-BioNTech (Comirnaty pediátrica) contra a covid-19 administradas a crianças de 5-11 anos de idade no Brasil.

### Variáveis

A variável principal do estudo foi a taxa de cobertura vacinal, obtida pela razão entre o número de indivíduos que recebeu a segunda dose e a população-alvo, multiplicada por 100 para expressar o resultado em porcentagem (13). 

As taxas foram calculadas mensalmente, o que totalizou 24 pontos de análise, entre janeiro de 2022 e dezembro de 2023. A população-alvo utilizada como denominador foi baseada nas crianças de 5-11 anos elegíveis, conforme os critérios estabelecidos pelo Programa Nacional de Imunizações. 

Consideraram-se as diretrizes do Plano Nacional de Operacionalização da Vacinação Contra a Covid-19 (15). A vacinação foi escalonada, com a priorização inicial de crianças com comorbidades, indígenas e quilombolas e, posteriormente, crianças sem comorbidades, nas seguintes faixas etárias em ordem decrescente: 10-11 anos, 8-9 anos, 6-7 anos e 5 anos (15). 

A base de dados utilizada neste estudo disponibilizou o total populacional com a consideração desse escalonamento, a fim de garantir a adequação do denominador conforme as fases de elegibilidade (14,15).

### Fonte de dados e mensuração

Os dados de vacinação e da população-alvo foram extraídos da plataforma Vacinômetro, fornecida pelo Ministério da Saúde (14).

Essa plataforma foi desenvolvida pelo Departamento de Monitoramento, Avaliação e Disseminação de Informações Estratégicas em Saúde, vinculado à Secretaria de Informação e Saúde Digital, em colaboração com o Departamento de Imunização e Doenças Imunopreveníveis da Secretaria de Vigilância em Saúde e Ambiente. 

A fonte de dados da base cartográfica (malhas territoriais/arquivos espaciais) utilizada para a confecção dos mapas coropléticos foi obtida na sua versão de 2021 (16). Todos os dados utilizados na pesquisa, referentes ao período de janeiro de 2022 a dezembro de 2023, foram coletados em setembro de 2024.

### Métodos estatísticos

Os dados extraídos foram consolidados no software Microsoft Excel versão 365. As taxas de cobertura vacinal foram calculadas mensalmente para cada UF e macrorregião brasileira entre janeiro de 2022 e dezembro de 2023.

As taxas foram exportadas para regressão segmentada (Joinpoint Regression Program versão 5.2.0). O software foi amplamente utilizado para a análise de tendências e identificação de pontos de inflexão em séries temporais, especialmente em estudos epidemiológicos (17). Ele permitiu identificar períodos de alterações significativas nas taxas e quantificar a magnitude dessas mudanças ao longo do tempo (18).

A variável dependente (Y) foi a taxa de cobertura vacinal mensal da vacina administrada à população de 5-11 anos, enquanto o mês foi considerado a variável independente (X). 

A análise foi realizada por meio de regressão segmentada com transformação logarítmica, com a utilização do método de permutação de Monte Carlo para verificar se múltiplos segmentos explicam melhor o modelo do que uma única reta. 

Calculou-se a variação percentual mensal (VPM) com intervalo de confiança de 95% (IC95%). Ao final da análise, a tendência geral de cada série foi sintetizada pela variação percentual média mensal (VPMM) entre os segmentos, com seus respectivos IC95% (20).

O modelo foi ajustado partindo de zero (um segmento) até quatro pontos de inflexão (três segmentos), com a consideração de nível de significância de 5% para testar a hipótese de que as VPM são estatisticamente diferentes de zero. Resultados com p-valor<0,005 foram considerados significativos, enquanto coeficientes (VPMM/VPM) negativos indicaram tendência decrescente, e positivos, crescente (21).

Utilizou-se o software RStudio, versão 2024.09 (22), para a demonstração gráfica e comparativa. Mapas coropléticos foram construídos com o software QGIS, versão 3.34.8 (23), para visualizar a distribuição das taxas de cobertura vacinal e identificar variações geográficas, segundo a UF e a macrorregião. Foi calculada a média aritmética simples das taxas mensais de cobertura vacinal, com a consideração da soma das taxas mensais e da sua posterior divisão pelo período completo (24 meses).

## Resultados

Os resultados deste estudo indicaram que, ao longo de 2022, a taxa de cobertura vacinal infantil da segunda dose contra a covid-19 variou entre as regiões brasileiras, Centro-Oeste, Nordeste, Norte, Sudeste e Sul, além do total nacional. Essa taxa atingiu seu pico em março em todas as regiões do Brasil. O Nordeste apresentou a maior taxa, com 26,0%, enquanto o Centro-Oeste e o Norte atingiram picos de 18,0% e de 16,0%. O Sudeste e o Sul registraram picos de 10,0% e de 8,0%. Após março, houve declínio gradual nas taxas em todas as regiões. Em comparação às outras, as regiões Sudeste e Sul mantiveram cobertura vacinal mais elevada após o pico inicial (Figura 1).

**Figura 1:** Cobertura vacinal da segunda dose contra covid-19 (%) em crianças de 5-11 anos. Brasil e macrorregiões, 2022 (n=57.506.832)

Em 2023, a cobertura vacinal infantil da segunda dose contra a covid-19 no Brasil permaneceu abaixo de 1,0% em todos os meses e regiões, com exceção da região Sul, que ultrapassou essa marca em março, atingindo 10,0%. As regiões Norte e Nordeste apresentaram comportamento semelhante, mas com taxas menores, que variaram entre 0,5% e 0,8%, em comparação ao Sul e ao Sudeste, que apresentaram taxas mais elevadas, com o Sudeste alcançando 7,5% em março. O Centro-Oeste se aproximou da média nacional em vários pontos, com taxas que variaram de 0,9% a 1,0%, o que reforçou um padrão intermediário. De forma geral, todas as regiões mantiveram níveis de cobertura vacinal baixos, especialmente se comparados ao ano anterior.

A análise da tendência de regressão segmentada indicou estabilidade na variação percentual média mensal (VPMM -8,3%; IC95% -21,6; 0,3) na cobertura da segunda dose da vacina contra a covid-19 no público de 5-11 anos entre 2022 e 2023. 

A análise regional dos dados revelou padrões distintos entre as macrorregiões brasileiras. Observou-se tendência estatisticamente estável na cobertura vacinal na região Nordeste (VPMM 5,2%; IC95% -0,4; 11,4), o que indicou ausência de crescimento significativo. No Norte, observou-se tendência crescente (VPMM 5,7%; IC95% 1,8; 13,0). No Centro-Oeste (VPMM 2,0%; IC95% -0,8; 6,7) e no Sul (VPMM 5,8%; IC95% -0,1; 13,3), a tendência foi estável. No Sudeste, a tendência foi decrescente (VPMM -8,30%; IC95% -11,2; -5,9), o que contrariou o padrão observado nas demais regiões (Tabela 1).

**Tabela 1:** Variação percentual média mensal (VPMM) e intervalo de confiança de 95% (IC95%) da cobertura vacinal da segunda dose contra covid-19 em crianças de 5-11 anos. Brasil, macrorregiões e unidades federativas, 2022-2023 (n=24)

Variável	Média cobertura vacinal 2022 (%)	Média cobertura vacinal 2023 (%)	VPMM	Inferior (IC95%)	Superior (IC95%)	p-valor	Tendência
Nordeste	4,4	0,4	5,2	-0,4	11,4	0,066	Estável
Alagoas	3,5	0,2	28,1	2,2	58,9	0,025	Crescente
Bahia	4,2	0,4	4,0	-1,6	11,5	0,143	Estável
Ceará	5,0	0,5	5,2	0,5	14,6	0,025	Crescente
Maranhão	3,0	0,4	7,1	-6,3	18,9	0,094	Estável
Paraíba	4,9	0,6	11,7	-0,7	16,4	0,068	Estável
Pernambuco	2,6	0,2	11,8	-8,4	34,2	0,107	Estável
Piauí	6,0	0,5	3,2	-1,8	10,2	0,237	Estável
Rio Grande do Norte	4,5	0,3	0,1	-3,3	3,9	0,944	Estável
Sergipe	5,0	0,6	10,2	4,7	17,6	0,032	Crescente
Norte	2,8	0,5	5,7	1,8	13,0	0,002	Crescente
Acre	2,7	0,5	23,4	8,4	47,4	0,010	Crescente
Amapá	3,5	0,9	12,1	5,5	24,5	<0,001	Crescente
Amazonas	3,5	1,0	18,2	10,0	43,3	0,010	Crescente
Pará	2,7	0,4	-4,7	-10,2	-0,6	<0,024	Decrescente
Rondônia	1,9	0,3	0,6	-3,8	6,8	0,767	Estável
Roraima	1,4	0,4	16,6	10,0	31,5	0,001	Crescente
Tocantins	2,1	0,2	8,1	-6,3	24,6	0,106	Estável
Centro-Oeste	3,2	0,3	2,0	-0,8	6,7	0,194	Estável
Distrito Federal	4,5	0,5	-0,0	-4,1	5,4	0,835	Estável
Goiás	3,3	0,3	8,8	3,0	18,5	0,001	Crescente
Mato Grosso	2,3	0,2	3,5	-2,3	12,1	0,208	Estável
Mato Grosso do Sul	3,1	0,3	-4,7	-8,3	-0,2	0,040	Decrescente
Sudeste	5,3	0,3	-8,3	-11,2	-5,9	<0,001	Decrescente
Rio de Janeiro	4,2	0,2	-4,2	-7,9	-0,7	0,027	Decrescente
Espírito Santo	4,2	0,6	8,0	-0,6	22,3	0,064	Estável
Minas Gerais	5,1	0,3	5,5	-1,3	15,2	0,081	Estável
São Paulo	5,9	0,3	12,7	-15,8	-10,4	<0,001	Crescente
Sul	4,2	0,3	5,8	-0,1	13,3	0,058	Estável
Paraná	3,4	0,2	3,9	-2,4	11,7	0,230	Estável
Santa Catarina	2,8	0,1	3,6	-4,1	10,7	0,228	Estável
Rio Grande do Sul	4,4	0,3	9,3	3,1	16,2	0,001	Crescente
Brasil	1,9	0,1	-8,3	-21,6	0,3	0,057	Estável

Destacaram-se 10 UF (37,0%) com tendências crescentes: Alagoas (VPMM 28,1%; IC95% 2,2; 58,9), Acre (VPMM 23,4%; IC95% 8,4; 47,4), Amazonas (VPMM 18,2%; IC95% 10,0; 43,3), Roraima (VPMM 16,6%; IC95% 10,0; 31,5), Amapá (VPMM 12,1%; IC95% 5,5; 24,5), Sergipe (VPMM 10,2%; IC95% 4,7; 17,6), Rio Grande do Sul (VPMM 9,3%; IC95% 3,1; 16,2), Goiás (VPMM 8,8%; IC95% 3,0; 18,5), Tocantins (VPMM 8,1%; IC95% -6,3; 24,6) e Ceará (VPMM 5,2%; IC95% 0,5; 14,6). Três UF apresentaram tendência decrescente (11,1%): Mato Grosso do Sul (VPMM -4,7%; IC95% -8,3; -0,2), Pará (VPMM -4,7%; IC95% -10,2; -0,6) e Rio de Janeiro (VPMM -4,2%; IC95% -7,9; -0,7). As outras 14 UF apresentaram tendência estável ao longo do período (51,9%).

**Figura 2:** Cobertura vacinal da segunda dose contra covid-19 (%) em crianças de 5-11 anos. Brasil e macrorregiões, 2023 (n=57.506.832)

Durante a análise da VPM, houve variações marcantes na cobertura vacinal ao longo do tempo nas diferentes regiões do Brasil, com um padrão comum de crescimento inicial seguido por quedas acentuadas e posterior estabilização (Figura 3). No período de 24 meses, as maiores variações ocorreram nos primeiros meses de 2022, com aumento expressivo seguido de rápida redução nas taxas, que se estabilizaram em baixos níveis até 2023.

**Figura 3:** Variação percentual mensal (VPM), Mudança Percentual Anualizada (MPC) e intervalo de confiança de 95% da cobertura vacinal da segunda dose contra covid-19 em crianças de 5-11 anos: (A) Brasil; (B) Centro-Oeste; (C) Sul; (D) Sudeste; (E) Norte; (F) Nordeste. Brasil e macrorregiões, 2022-2023 (n=24)

No Brasil, o primeiro segmento apresentou VPM elevada de 603,0% (IC95% 48,6; 2.439,5) de janeiro a março de 2022, seguido pela desaceleração acentuada de 24,4% (IC95% -30,7; -22,1) ao longo de todo o restante da série temporal. No Norte e no Nordeste, os valores iniciais de VPM foram altos, com 791,4% (IC95% 540,2; 1.929,1) no Norte e 1.557,6% (IC95% 825,3; 3.275,1) no Nordeste, seguidos por queda no Norte (-22,9%; IC95% -29,1; -20,1) e no Nordeste (-34,2%; IC95% -38,8; -31,1). Depois, no Norte, houve estabilidade nos últimos meses (mudança percentual anualizada [MPC] -7,5; IC95% -12,4; 1,3) e, no Nordeste, manutenção da queda (VPM -12,0%; IC95% -16,6; -7,3), embora com menor intensidade.

No Centro-Oeste, a VPM inicial também foi alta (875,1%; IC95% 690,8; 1.615,8), seguida de desaceleração gradual (VPM -28,9; IC95% -31,9; -27,2) e com menor ritmo ao final do seguimento (VPM -9,8%; IC95% -13,9; -4,1). No Sudeste, houve 4 regressões segmentadas, com aumento inicial de 349,4% (IC95% 293,3; 477,9), seguido por queda acentuada no terceiro e no sexto mês (VPM -45,5%; IC95% -48,6; -42,3) e do sexto ao décimo mês (VPM -27,5%; IC95% -33,9; -13,5), com estabilização ao final da série (MPC -12,6%; IC95% -22,3; 5,8). 

No Sul, observaram-se crescimento elevado (VPM 1.918,6%; IC95% 1.079,5; 4.821,8) e rápida desaceleração (VPM -38,6; IC95% -43,5; -35,4), com menor força ao final (VPM -11,1%; IC95% -16,5; -5,3), semelhante a outras regiões.

O Sudeste apresentou as maiores taxas de cobertura vacinal da segunda dose da vacina de covid-19 para crianças de 5-11 anos (61,0%-71,0%), seguido pelo Nordeste e pelo Sul, com taxas intermediárias (51,0%-61,0%). As menores taxas de cobertura vacinal foram registradas nas regiões Norte e Centro-Oeste. Na região Norte, as taxas variaram de 1,5% a 4,0%, enquanto na região Centro-Oeste, as taxas oscilaram entre 2,0% e 4,5%. Piauí e São Paulo registraram as maiores taxas (73,0%-83,0%) entre as UF, seguidos por Minas Gerais, Ceará, Pernambuco e Alagoas (63,0%-73,0%). Mato Grosso, Tocantins, Roraima e Rondônia apresentaram as menores taxas (23,0%-33,0%) (Figura 4).

**Figura 4:** Distribuição espacial das taxas de cobertura vacinal da segunda dose contra covid-19 (%) em crianças de 5-11 anos: (A) unidades federativas; (B) macrorregiões brasileiras. Brasil, 2022-2023 (n=57.506.832)

## Discussão

A análise da cobertura vacinal pediátrica contra a covid-19, com foco na segunda dose, no Brasil em 2022 e em 2023 revelou um cenário de significativa heterogeneidade geográfica e temporal, apesar da estabilidade nacional na VPMM. Observou-se crescimento acentuado da cobertura na região Norte, com destaque para as UF do Acre e do Amazonas. Em contrapartida, a região Sudeste e algumas UF específicas (Rio de Janeiro, Mato Grosso do Sul e Pará) apresentaram tendência declinante. 

Este estudo apresentou limitações inerentes ao uso de dados secundários, que podem estar sujeitos a inconsistências, incompletudes e possíveis vieses de registro, o que pode ocasionar divergências em relação aos valores reais de cobertura vacinal. A implementação escalonada da vacinação pediátrica no Brasil, embora considerada no ajuste do denominador populacional, pode ter influenciado as taxas iniciais e afetado a comparabilidade temporal entre as UF. Por se tratar de estudo observacional descritivo com dados agregados, não foi possível realizar inferências causais ou análises em nível individual.

Os achados de heterogeneidade geográfica na cobertura vacinal pediátrica corroboram outras identificações de padrões distintos ao longo do tempo e entre os territórios (15). Historicamente, a região Norte apresenta as menores coberturas, seguida por áreas do Nordeste e do Centro-Oeste (10,24). Até dezembro de 2022, foram identificadas nove UF com cobertura vacinal abaixo de 70% (localizadas nas regiões Norte e Nordeste), reconhecidas por maiores vulnerabilidades socioeconômicas (4).

A baixa cobertura nessas regiões tem sido relacionada a fatores como menor índice de desenvolvimento humano, dificuldades de acesso aos serviços de saúde, grandes extensões territoriais e baixa densidade populacional, especialmente em municípios da região Norte (24,4,30). Essas desigualdades estruturais contribuem para flutuações nos indicadores ao longo do tempo e refletem disparidades históricas no acesso e na administração de vacinas, em contraste com as regiões Sul e Sudeste, que tradicionalmente apresentam melhores índices de cobertura vacinal (30).

As evidências de estabilidade ou de declínio em certas regiões reforçam a urgência de compreender os fatores limitantes da adesão vacinal. A hesitação vacinal, frequentemente exacerbada pela disseminação de desinformação, constitui obstáculo significativo (10). Fatores como preferências políticas, consumo de determinadas mídias e características sociodemográficas dos pais (idade, renda, escolaridade, afiliação religiosa) influenciam diretamente a aceitação da vacina, com menor adesão observada entre grupos alinhados a posturas antivacina (29).

Apesar desses desafios, é fundamental reforçar a importância da vacinação pediátrica. A imunização reduz os riscos de casos graves, hospitalizações e óbitos por covid-19, inclusive de complicações como a síndrome inflamatória multissistêmica pediátrica e a covid-19 longa (25-28). A vacinação contribui para a imunidade coletiva, a proteção de grupos vulneráveis e o retorno seguro às atividades cotidianas, o que alivia a carga sobre os sistemas de saúde. A integração da vacinação contra a covid-19 com programas de imunização de rotina e uso de incentivos, como o aconselhamento por profissionais de saúde, ajuda a impulsionar a adesão (24).

A análise temporal demonstrou um padrão de crescimento rápido da imunização infantil no início da campanha de vacinação contra a covid-19 em 2022, seguido por queda acentuada e estabilização em baixos níveis ao longo de 2023. Esse comportamento pode estar relacionado ao início da campanha, que ocorreu sob a coordenação do Ministério da Saúde, conforme as diretrizes do Plano Nacional de Operacionalização da Vacinação contra a covid-19. A estratégia foi de abrangência nacional, com previsão de distribuição de doses às UF e aos municípios, porém sua implementação apresentou variações de acordo com a capacidade logística local, a adesão social e a estrutura da atenção primária em saúde, o que resultou em diferentes ritmos de cobertura entre as UF (14,15). 

É importante que existam ações mais concretas para reverter as baixas coberturas vacinais em 2022 e 2023, como o fortalecimento de campanhas educativas regionalizadas, a ampliação do horário de funcionamento das salas de vacina, o incentivo ao envolvimento das escolas e a maior integração entre entes federativos, a fim de garantir oferta e acesso equitativo à vacinação infantil (4,6,24,29,30).

Este estudo evidenciou um cenário complexo e heterogêneo da cobertura vacinal pediátrica, com foco na segunda dose, contra a covid-19 no Brasil, com tendências regionais divergentes e padrão temporal de desaceleração após o avanço inicial. Tais achados são de grande relevância para o planejamento de políticas públicas, que devem considerar as variações para fortalecer a imunização infantil e assegurar a proteção dessa população vulnerável.

## Data Availability

Os bancos de dados usados para análise desta pesquisa estão disponíveis em: https://doi.org/10.6084/m9.figshare.29251784. Os dados de vacinação e de população-alvo foram extraídos da plataforma Vacinômetro, fornecida pelo Ministério da Saúde: https://infoms.saude.gov.br/extensions/SEIDIGI_DEMAS_Vacina_C19/SEIDIGI_DEMAS_Vacina_C19.html.
